# A Rare Case of Multidrug-Resistant Tuberculosis Affecting the Pleura

**DOI:** 10.7759/cureus.21690

**Published:** 2022-01-28

**Authors:** Khalid Jamal, Muhammad Imran, Shah Hassan Khan, Abdul Muneem, Muhammad Salman Khan

**Affiliations:** 1 Pulmonology, Saidu Teaching Hospital, Swat, PAK; 2 Pulmonary and Critical Care, Lady Reading Hospital, Peshawar, PAK; 3 Pulmonology and Critical Care, COVID-19 Hospital, Peshawar, PAK; 4 General Practice, Medical Emergency and Resilience Foundation, Peshawar, PAK

**Keywords:** multidrug-resistant tb, line probe assays (lpa), isoniazid resistance, rifampicin resistance, mdr-tb, tuberculosis, pleural effusion, extra-pulmonary tuberculosis

## Abstract

The majority of cases with tuberculous pleuritis have negative acid-fast bacilli (AFB) on smear microscopy, making the diagnosis difficult. This case report is based on the successful diagnosis and management of an extra-pulmonary (EP) multidrug-resistant tuberculosis (MDR-TB) patient with a history of lymphoma. Initial tests revealed a right-sided pleural effusion and thickening of the pleura. The closed pleural biopsy, pleural fluid histopathology, culture, and drug sensitivity testing (DST) report revealed *Mycobacterium tuberculosis* with isoniazid and rifampicin resistance. Based on the DST report, the patient was labeled as a case of MDR-TB and successfully managed with an individualized drug-resistant TB (DR-TB) regimen. With initial negative microscopy and GeneXpert MTB/RIF (Sunnyvale, CA: Cepheid Inc.) reports, this case demonstrated that DR-TB could exist even in the absence of risk factors. Furthermore, it also unveils the importance of line probe assays (LPAs) and culture in identifying MDR-TB. Lymphocytic/exudative pleural effusions and pleural biopsy specimens should be subjected early on to investigations like Xpert/MTB RIF, cultures, and genotypic DST to timely diagnose and treat DR-TB.

## Introduction

Pleura is one of the most common sites of extra-pulmonary tuberculosis (EP-TB), which occurs in approximately 5% of people infected with *Mycobacterium tuberculosis* (MTB) [1]. In TB-endemic areas, however, the prevalence reaches up to 30% [2]. Diagnosis is complex, with 48-96% of tuberculous pleural effusions being negative by acid-fast bacilli (AFB) microscopy and culture. Thoracentesis is frequently performed and reveals an exudative, lymphocytic pleural effusion in more than 90% of patients, whereas direct investigation reveals AFB in less than 10% of patients [3]. More invasive diagnostic procedures are usually required because of the low specificity and sensitivity of AFB staining and cultures. We present a case that demonstrates the clinical presentation and diagnostic approaches of primary extra-pulmonary MDR-TB in an HIV-negative older adult with right-sided pleural effusion.

## Case presentation

**Figure 1:**
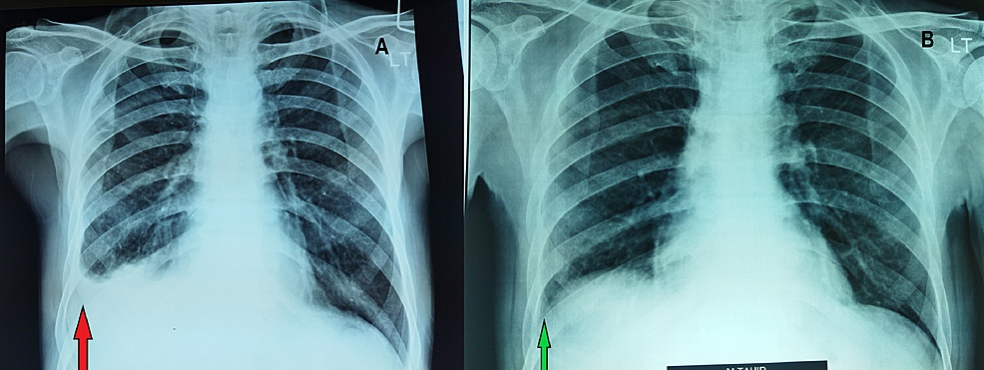
Pre- and post-treatment chest x-ray PA view. (A) Pre-treatment CXR-PA view with blunting of right costophrenic angle (green arrow), which is a sign of effusion. (B) The effusion has resolved completely, with a normal costophrenic angle (red arrow) in the follow-up CXR-PA view at sixth month of treatment. PA: posteroanterior; CXR: chest x-ray

A general practitioner (GP) referred a 50-year-old male patient to Programmatic Management of Drug-Resistant Tuberculosis (PMDT) unit Swat, Pakistan, with chief complaints of fever, anorexia, fatigue, and a 15 kg weight loss in one-month duration. The patient also attested to right-sided chest pain. On examination, he was ill-looking, pale, and had decreased air entry on the right side with dull percussion notes. The temperature was 101°F and blood pressure was 110/70 mmHg. He was treated with antibiotics and analgesics for lower respiratory tract infection without proper workup. A chest x-ray posteroanterior (PA view) was performed which showed blunting of right costophrenic angle as shown in Figure 1, panel A.

He has had a significant past medical history. Fifteen years before his current presentation, the patient presented to the medicine outpatient department (OPD) with generalized lymphadenopathy. Cervical lymph nodes were biopsied, and the histopathology showed chronic non-specific inflammation. He was started empirically on antitubercular therapy (ATT). After taking ATT for three months, the non-resolving cervical lymph nodes were re-excised, which showed Hodgkin's lymphoma. The patient was treated successfully as a case of lymphoma with chemotherapy and his symptoms resolved entirely.

An ultrasound-guided diagnostic pleural tap indicated a right-sided pleural effusion, as shown in Figure 1, panel A. Two pleural fluid samples and a closed pleural biopsy were collected using Abram's needle, followed by therapeutic aspiration of the fluid. One pleural fluid sample was submitted to the National Reference Laboratory (NRL) for GeneXpert (Sunnyvale, CA: Cepheid Inc.), AFB culture, and genotypic drug sensitivity testing (DST). The other sample was sent to the hospital laboratory for routine examination, conventional cytology, Gram staining, and culture. It was found to be a lymphocytic exudative effusion. No malignant cells were found. Moreover, bacterial cultures were negative after 48 hours, and histopathology was inconclusive. NRL provided genotypic DST utilizing the MTBDR-plus (Nehren, Germany: Hain Lifescience GmbH) line probe assay (LPA), which showed a rpoB 530-533 rifampicin mutation, and an isoniazid mutation in KatG-S315T1, and the patient was labeled as having extra-pulmonary MDR-TB (Table 1). Both fluoroquinolones and second-line injectable's results were indeterminate.

**Table 1:** Genotypic DST (MTBRsl-plus) result. Result of LPA MTBDR-plus, there was resistance inferred and detected to rifampicin and Isoniazid, respectively. Resistance to fluoroquinolones and second-line injectable drugs were reported as indeterminate. LPA: line probe assay; MTBDR: *Mycobacterium tuberculosis* drug-resistant; AFB: acid-fast bacilli; DST: drug sensitivity testing

Specimen type	Appearance	Volume	AFB microscopy	Genotypic drug susceptibility test performed on LPA MTBDR-plus				
Pleural fluid	Turbid	5.0 ml	AFB smear-negative	Drug	Resistance predicted	Resistant Gene	Mutation detected	Results interpretation
				Rifampicin	Resistance inferred	rpoB	530-533	Rifampicin is not effective
				Isoniazid	High-level resistance detected	katG	S315T	Isoniazid is unlikely to be effective even at high dose
				Fluoroquinolones	Indeterminate			
				Second-line injectable drugs	Indeterminate			

The patient was started on an individualized treatment regimen. Initial doses were, 1000 mg levofloxacin, 600 mg linezolid, 100 mg clofazimine, 750 mg cycloserine, 1600 mg pyrazinamide, 750 mg ethionamide, and 150 mg Vita-6. According to his psychiatric evaluation, his protracted sickness had left him somewhat depressed. He was motivated to cooperate with treatment and began psychotherapy. We employed incremental muscular relaxation, distraction, and deep breathing.

He was seen biweekly for the first month, then monthly. Each appointment included a psychological examination, which included a recap of the prior session. After three months of medication, the right-sided pleural effusion vanished, and the patient's chest x-ray and clinical condition remarkably improved (Figure 1, panel B).

The initial phase of linezolid and ethionamide was completed, and they were stopped at sixth month, while the rest of the therapy was continued for 12 more months. The patient had no recurrence of his pleural effusion till the last follow-up. The patient's closed contacts were counseled and screened regularly for any evidence of TB. No one was found positive or having any symptoms of TB.

## Discussion

TB is one of the major public health issues in Pakistan; according to the Global TB Report, Pakistan ranks fifth among 30 high-burden nations for drug-sensitive TB (DS-TB) and fifth for drug-resistant TB (DR-TB) [4]. The causes of DR-TB include poor adherence to treatment, the use of low-quality medicine in the private sector, diagnostic delays, unsupervised therapy, and inadequate follow-up [5]. According to reports, DR-TB was discovered in 3-4% of new pulmonary cases and 18-21% of previously treated patients [6]. Furthermore, there is scarce data on extra-pulmonary drug-resistant tuberculosis in the literature. Table 2 shows the published cases according to PubMed Central. Yadav and Rawal reported similar cases of MDR-TB in the pediatric age group with pleura involvement [7]. As shown in Table 2, Anastasakos et al. found 103 cases of tubercular pleural effusion (TPE), with 11 showing DR-TB patterns [8]. Among them three showed resistance to isoniazid (INH), three were labeled as MDR-TB, one was extended drug-resistant TB (XDR-TB) and, seven showed other patterns of resistance. As a result, we have reported the first adult case of primary MDR-TB manifesting as pleural effusion, which has not previously been reported.

**Table 2:** Drug-resistant tubercular pleural effusion: published research. DR-TB: drug-resistant tuberculosis, MDR: multidrug-resistant tuberculosis, XDR: extended drug-resistant tuberculosis; INH: isoniazid

S.No	Authors	Year of publication	Journal	Sample size	Resistance pattern
1	Yadav and Rawal [7]	2016	Transnational Pediatrics	1	Rifampicin and INH
2	Anastasakos et al. [8]	2017	The International Journal of Tuberculosis and Lung Disease	Total: 4391, pleural TB: 103, and DR-TB pleural effusion: 11	MDR: 3, XDR: 1, others: 7
3	Olson [9]	2019	Open Forum Infectious Diseases	1	INH

Even though our patients' HIV testing was negative, all patients with extra-pulmonary TB should be screened for HIV infection because positive HIV status has been linked to increased risk of both DS- and DR-TB [10]. Furthermore, in the case of EP-TB infections, where the diagnosis may be based solely on clinical and early laboratory results without final microbiological confirmation, a higher index of suspicion is usually required [11]. Diagnosis of any form of tuberculosis, particularly extra-pulmonary DR-TB, can be difficult for physicians at times. As in our case, the diagnosis was delayed because of negative smear microscopy and Xpert MTB/RIF results. Likewise, using biomarkers to aid in diagnosing increases the risk of incorrectly treating DS-TB as DR-TB [12]. As discussed, diagnosing MDR-TB in non-pulmonary samples is challenging and leads to high mortality and morbidity. Early identification of the TB strain through LPA and culture resulted in the successful management of the case and reduced the risk of complications.

## Conclusions

Clinical factors and chest radiographic findings associated with MDR-TB should prompt physicians to perform Xpert MTB/RIF and DST sooner, as the patient is at risk of developing pulmonary or extra-pulmonary DR-TB if an early diagnosis is not made. As a result, we recommend using GeneXpert MTB/RIF as a primary diagnostic technique on most tissue specimens in resource-limited settings. Moreover, cultures should be done in all cases where DR-TB is suspected, as, at times, rapid tests like Xpert MTB/RIF and microscopy are negative in cases of EP-TB.
